# Computational medical imaging and hemodynamics framework for functional analysis and assessment of cardiovascular structures

**DOI:** 10.1186/s12938-017-0326-y

**Published:** 2017-03-21

**Authors:** Kelvin K. L. Wong, Defeng Wang, Jacky K. L. Ko, Jagannath Mazumdar, Thu-Thao Le, Dhanjoo Ghista

**Affiliations:** 1 School of Medicine, University of Western Sydney, Campbelltown, Sydney, NSW 2560 Australia; 2 Department of Imaging and Interventional Radiology, The Chinese University of Hong Kong, Shatin, New Territories Hong Kong; 3 Centre for Biomedical Engineering and School of Electrical and Electronics Engineering, University of Adelaide, Adelaide, SA 5005 Australia; 4 National Heart Centre, Mistri Wing, 17 Third Hospital Avenue, Singapore, 168752 Singapore; 5 University 2020 Foundation, Northborough, MA 01532 USA; 6 School of Medicine, Western Sydney University, Locked Bag 1797, Penrith, NSW 2751 Australia

**Keywords:** Hemodynamics performance indicators, Medical imaging, Medical image reconstruction, Computer simulation, Cardiovascular disease

## Abstract

Cardiac dysfunction constitutes common cardiovascular health issues in the society, and has been an investigation topic of strong focus by researchers in the medical imaging community. Diagnostic modalities based on echocardiography, magnetic resonance imaging, chest radiography and computed tomography are common techniques that provide cardiovascular structural information to diagnose heart defects. However, functional information of cardiovascular flow, which can in fact be used to support the diagnosis of many cardiovascular diseases with a myriad of hemodynamics performance indicators, remains unexplored to its full potential. Some of these indicators constitute important cardiac functional parameters affecting the cardiovascular abnormalities. With the advancement of computer technology that facilitates high speed computational fluid dynamics, the realization of a support diagnostic platform of hemodynamics quantification and analysis can be achieved. This article reviews the state-of-the-art medical imaging and high fidelity multi-physics computational analyses that together enable reconstruction of cardiovascular structures and hemodynamic flow patterns within them, such as of the left ventricle (LV) and carotid bifurcations. The combined medical imaging and hemodynamic analysis enables us to study the mechanisms of cardiovascular disease-causing dysfunctions, such as how (1) cardiomyopathy causes left ventricular remodeling and loss of contractility leading to heart failure, and (2) modeling of LV construction and simulation of intra-LV hemodynamics can enable us to determine the optimum procedure of surgical ventriculation to restore its contractility and health This combined medical imaging and hemodynamics framework can potentially extend medical knowledge of cardiovascular defects and associated hemodynamic behavior and their surgical restoration, by means of an integrated medical image diagnostics and hemodynamic performance analysis framework.

## Application of computational fluid dynamics in cardiovascular health assessment

The modern medical imaging community in practice is associated with a landscape of non-invasive imaging, image processing and cardiovascular analyses components, which constitute the traditional means of assessing cardiovascular system performance. The present-day cardiologists diagnose patients with diseases via visual observations of the heart and arterial system, from well-established clinical imaging such as echocardiography, magnetic resonance imaging/angiography, chest radiography, and computed tomography, by which they provide their expert opinions on the appropriate medical treatment. In recent decades, high fidelity multi-physics computational platforms that yield cardiovascular hemodynamics patterns have been developed, are researched, and can be integrated as a support tool into the existing medical imaging systems to generate a more precision-based patient-specific diagnosis of cardiovascular conditions. This kind of integrated high-performance computational platform generates the relevant hemodynamics mechanics to support the medical imaging-based diagnosis by quantification of hemodynamic patterns and parametric values for decision making and generation of expert opinions for surgical treatment.

Cardiac disease can introduce various cardiovascular defects in the human body, and can be examined using modern imaging diagnostics. Herein, we briefly assess the abnormality of some of these defects—such as septal defects, myocardial fibrillations, arrhythmias, heart valve failure and other heart diseases can be assessed for degrees of abnormality, and also evaluate the post-surgical treatment outcomes of some of these defects. Cardiac flow fields that are obtained from clinical ultrasound-based or phase-contrast MR image scan sequences can be analyzed, and interesting flow phenomena relating to the operation of cardiac structures such as heart valves can be discovered. Nevertheless, qualitative assessment of the cardiovascular defects does not give a proper evaluation of the underlying cause and effect, as well as comparison of the healthy and abnormal conditions. Appropriate hemodynamic analysis based parameters need to be used for quantitative studies. For instance, hemodynamic analysis of the heart structure has been investigated in the vascular network based on localized wall shear stress (WSS) regions, since it is known that the WSS has an implication on atherosclerosis [1]. Quantification of blood flow shear stress in various pathologies such as atherosclerotic arteries, the aorta, and in the coronary arteries of the heart can reveal how WSS influence atherogenesis. We note that this WSS analysis can also be extended to the investigation of artificial cardiac assist devices, such as coronary stents [2–4], ventricular assist devices [5, 6], and heart valves [7, 8]. In particular, myocardial diseases occurrence in cardiovascular arteries can be examined from the hemodynamics perspective. Importantly, the applications derived from combining medical imaging and computational processing with computational hemodynamic analysis, as reviewed in this paper, can be used to investigate cardiac health in relation to atherosclerosis.

In the context of atherosclerotic arteries, the superficial carotid bifurcation is an ideal target for non-invasive imaging via ultrasound (US), magnetic resonance imaging (MRI) or computed tomographic (CT) imaging, and is hence used as a case study for our review in this paper. The angiographic techniques such as magnetic resonance angiography, X-ray angiography, etc. are often commonly used in examining atherosclerosis. For example, cineangiograms are used by medical doctors to visually examine the degree of stenosis in carotid bifurcations or coronary arteries. However, they fail to explain the underlying cause of the problem from the blood flow perspective. It can be demonstrated that the plaque ulceration is related to the existence of high wall shear stress (WSS) at the upstream region of the plaque [9]. In this regard, a serial MRI-based study based on carotid artery plaques has proved that the regions exposed to low WSS and low wall stresses are most prone to develop atherosclerotic plaques [10]. There are also efforts to determine the influence of local risk factors, such as time-averaged wall shear stress (TAWSS) and oscillatory shear index (OSI) in atherosclerosis, in addition to the surrogate geometric markers of disturbed flow [11]. The sensitivity of these physiologically relevant parameters to the arterial inlet and outlet boundary conditions may also be investigated by image-based hemodynamics studies [12, 13].

From the perspective of how cardiac diseases (such as cardiomyopathy) affect the heart and its consequential remodelling, surgical ventricular restoration (SVR) addresses the issue of restoring the performance and health of remodeled left ventricles with large akinetic walls and dilated ventricles [14]. This type of procedure requires knowledge of the shape and size of the left ventricle (LV), as well as information on the intraventricular flow dynamics in order to design useful performance indicators for evaluation of heart pumping inefficiency. Computational hemodynamics comes into play when hemodynamics indicators, such as the resistance-to-filling and contractility information can be derived, based on the computed ventricular and myocardial wall volumes, intra-LV blood flow velocity fields and pressure gradients, after importing the geometry into a computational fluid dynamics (CFD) platform. At the initial stage of this pipeline, medical imaging of the heart is performed via dedicated scanners, and then fed into the image processing pipeline in order to achieve geometrical reconstruction of the cardiac chamber of interest, such as the LV. Slices that depict the relevant anatomy are selected based on the region of interest for analysis. Wall boundaries are defined and then used to build a geometrical deformable model of the LV. Mesh grids are generated and imported into a CFD platform to solve the transient flow fields. Due to the large ventricular wall movement, special considerations to the flow simulation needs to be taken care of, for example, the geometric conservation law needs to be used for unsteady flows with moving boundaries. Then, the Navier–Stokes equation is solved by using a high-performance computing to obtain the three-dimensional intra-cardiac flow field. Hemodynamics parameters assessing the performance of the LV are quantified. The information on healthy and normal patients can be input into a database, and statistical quantification based on a range of hemodynamics parameters can give support to medical doctors in terms of diagnosis, expert opinions on treatment, clinical advice, etc. [14, 15].

It may be of interest to experimentally determine the true flow fields which can be used as a more realistic version for analysis of the blood flow behavior in the heart. In addition, such information can also be used to validate the numerically computed flow fields. Functional medical imaging modalities are on the rise in terms of speed, accuracy and reliability in measurement of blood flow in the cardiovascular system. On the velocity-encoded (VENC) imaging technology front, phase contrast magnetic resonance imaging (PC-MRI) that is coupled with cardiac flow analysis [16, 17] gives rise to a system of direct visualization and analytical processes for describing flow behavior in cardiac chambers. The use of VENC MR imaging applied onto cardiac imaging of heart chambers can also enable a good assessment of hemodynamics that exists in the heart. As such, the topic of VENC MR imaging forms another short section of our review. It may be worthwhile pointing out that another common flow measurement modality is the ultrasound of the heart or echocardiography. It is well known that cardiac flow in the heart chambers generates spiraling motion or vortices [18, 19], and is essential for efficient heart operation and blood circulation [20]. All of this provides the basis for examining the flow behavior in cardiovascular structures in terms of cardiovascular health parameters or indicators.

## High-performance computing framework for hemodynamics assessment

### Overall view of framework

**Fig. 1:**
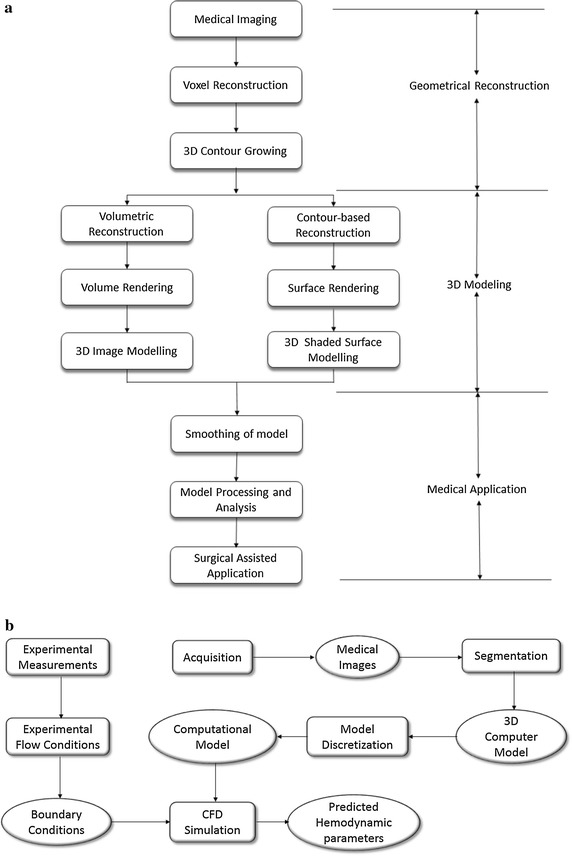
Procedural flow charts for medical imaging, geometrical reconstruction, and high-performance computing of hemodynamics parameters. This systematic approach performs medical imaging, which can facilitate the 3D model reconstruction (**a**) and computational fluid dynamics of cardiovascular structures (**b**). Data retrieval and anatomical reconstruction based on medical imaging generate a geometrical model of the cardiovascular structure. There are a few stages, such as volume or surface rendering to prepare these medical image slices for 3D geometrical construction of the organ or artery of interest. Then, using the anatomical model for surgical assisted applications, we may even implement the measurement of boundary conditions and using it to perform the numerical simulation based on the anatomical reconstructed model is performed. The predicted data from simulated flows are visualized, and useful hemodynamics indicators are extracted for analysis in the final stage

To developing an effective analytical computational simulation, the first step is to create the 3D computer model of the cardiovascular geometry. The detailed stages are presented in Fig. 1a. Medical imaging is a crucial stage if a patient-specific model is required. After the acquisition of multiple image slices across the organ or artery of interest, the generation of a 3D structure can be achieved by volumetric rendering or surface rendering. Eventually, the final geometry is appropriately processed for medical applications. Then, the overall methodology of combining experimentally measured flow boundary conditions with CFD model analysis to determine the hemodynamic parameters is depicted in Fig. 1b. Numerical studies of hemodynamic characteristics based on computational fluid dynamics (CFD) can be performed, based on an anatomically realistic cardiovascular model reconstructed from medical images. For image-based hemodynamics studies, the domain of interest is always truncated and taken out of the context of the entire circulatory system. The last stage would be the determination of hemodynamics flow patterns and indicators/parameters for cardiac health assessment.

The usefulness of experimental measurements is on the validation of the numerically simulated results and as a form of support for fluid mechanists to review the accuracy of their simulation platform. In addition, experimental data can also be used to determine the boundary conditions when setting the numerical framework, as illustrated in Fig. 1. In the case of such cardiovascular anatomies, velocity-encoded (VENC) phase contrast MRI or ultrasound (UTS), which are flow measurement techniques, are typically used to extract the flow values at the inlets and outlets of the cardiac anatomical structures to be used as boundary conditions [21–23]. The application of the inlet and outlet boundary conditions will considerably affect the numerical accuracy of the local risk factors such as TAWSS and OSI. Therefore, it is important to impose patient-specific inflow and outflow flow rates, based on the UTS or MRI measurements.

The system integration of the components outlined in the previous sections gives rise to a systematic approach for assessing the cardiac health condition of cardiovascular patients. This forms a generic approach to constructing a system for analyzing cardiac defects via examination of the flow variations derived from medical imaging and post-processing techniques. Let us visit each stage of the flow chart in the subsequent sub-sections.

### Medical imaging and anatomical reconstruction

This section reviews the medical imaging and visualization application, with unique quantification of some of the structural parameters, which can lead to a viable diagnostic system for evaluating cardiac related failures or health risk in patients. The ease and speed of these preparations are important considerations for medical doctors in order to quickly gain insights into the structures in the defective regions of the heart and to assist in strategizing surgical procedures, operations or artificial implantations.

This imaging and geometrical reconstruction system are vital because of the technical feasibility of using measured information from medical imaging to determine blood flow information. The key success is the result of implementing high-performance computing as a post-processing tool for the reconstructed geometry, which is produced by medical image reconstruction of images from medical scanners. This renders the encoding of velocity information redundant, which reduces scanning and processing time. Medical image reconstruction and high-performance computing open up many new opportunities for flow analysis concurrent to the examination of cardiac chambers, septal defects, and heart valve behavior. It may also be of interest to cardiologists and physiologists to provide information on the structural shape and size of the cardiac chambers in relation to the behavior of the flow in the heart. Inevitably, errors and noise appear in measured data. Numerical simulation framework and other mechanism can be adjusted in the event of errors. To reduce the effect of noise, we usually solve with least squares. Reducing distance between the geometrical entities (points, lines, planes, etc.) is minimizing geometric errors.

**Fig. 2:**
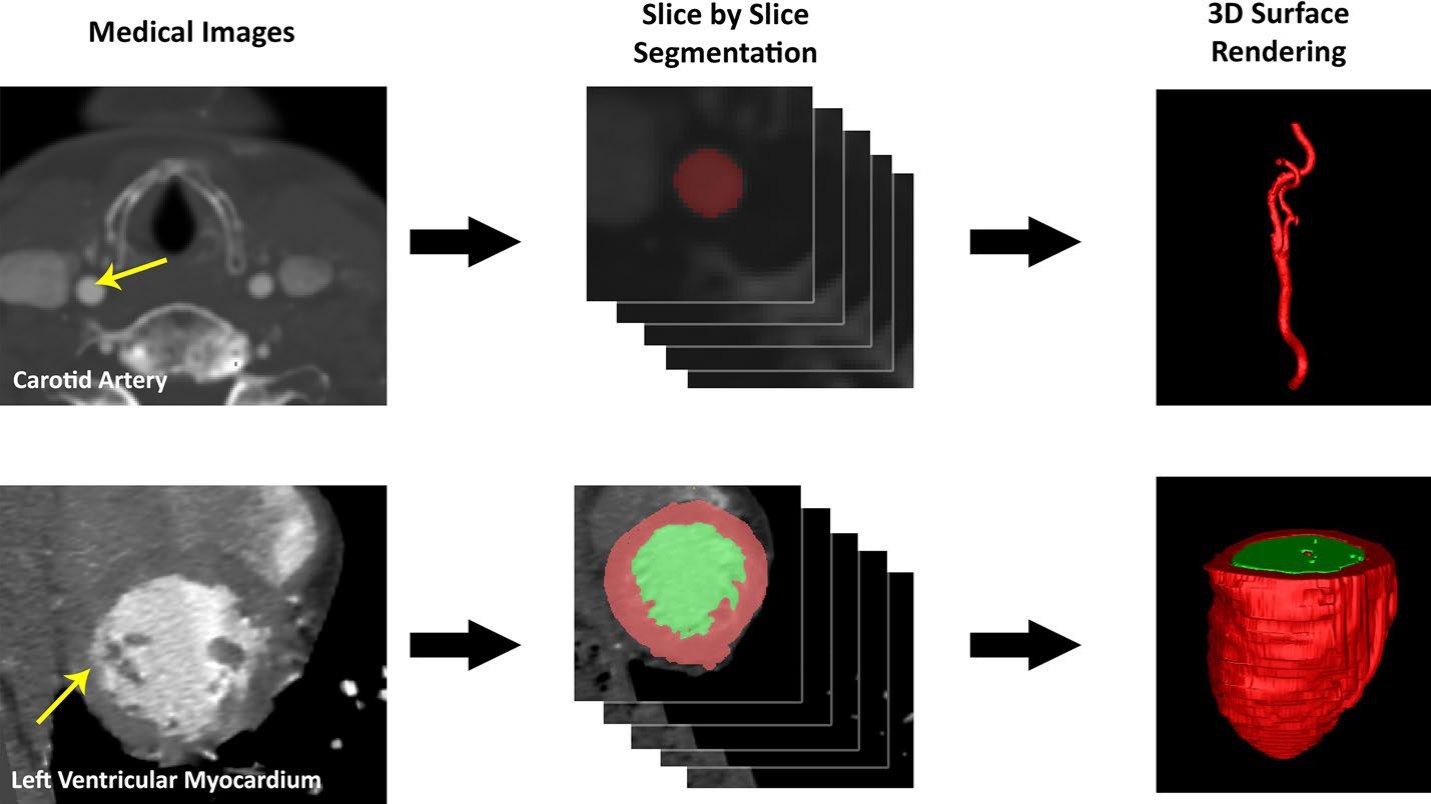
Medical image reconstruction of cardiovascular structures. Medical image reconstruction of cardiovascular structures. Computer tomographic angiography was carried out on the neck region of the patient whose carotid artery can be imaged at axial orientation for multiple slices. Segmentation based on the threshold of the blood vessel at various slices is performed in the initial stage. The segmented voxels can be grouped to form a three-dimensional anatomy and a mesh reconstruction based on the contours of these segmented regions is carried out (*up*). In a similar way, the left ventricle is imaged and ventricular chamber segmentation is performed. Then loft surface formation into a geometrical surface structure is enabled to give the anatomical model computationally (*down*)

Typically, the first objective is to map the cardiovascular structure of the heart chamber or arteries. From medical imaging modalities such as the ultrasound, MRI or CT scans of cardiac chambers such as the left ventricle (LV) myocardium, the wall boundary can be extracted and reconstructed to derive the dynamic LV geometry. In the example illustrated in Fig. 2, medical imaging using the steady-state free precession MRI protocol allows imaging to be registered within the cardiovascular structure at localized sections with specific depth and thickness. As demonstrated, the anatomical reconstruction of the LV endocardium into the chamber geometry, from the aforementioned steady-state free-precession MR images in the short-axis and long-axis scan orientation, can be performed by the triangulation algorithm [24].

Next, we examine the elements of the medical imaging and anatomical reconstruction platform that comprises of a generic scanning modality, an image processing and a geometrical boundary definition and modeling framework. It is essential to establish a network of sub-components such that each of the components has a role in preparing the anatomical reconstruction. The system layout of this computational reconstruction serves to produce the computational mesh of the cardiovascular structures, such as the cardiac chamber or the artery, that are obtained from medical imaging modalities such as MRI, CT, or ultrasound. The core functionality of this system is the visualization of anatomical structures and identification of their defects. Observation of the structural details generated by the system can assist in identifying stenosis or regions of critical plaque growth in the case of the atherosclerotic arteries, and septal defects in the case of discontinuity in the myocardium of the heart which can occur in the atrium or ventricle. It is worthwhile noting that cine-images of the cardiac chamber such as the atrium can be constructed and played via a multimedia tool to observe irregularity in beating and assist in diagnosis of atrial fibrillation. In addition, the geometrical mesh of the left ventricle (LV) output by the system can be fed into a simulation modeler, and solved for the intra-cardiac flow by using computational fluid dynamics to obtain useful flow behavior such as vortices or other interesting flow patterns within the chamber. Useful hemodynamics indicators can also be derived for these cardiovascular structures to support medical diagnosis of the cardiac condition.

From the computer architecture perspective, the procedures executed by the system of components are outlined as follows. After pre-processing, the medical images can be retrieved via a Receiver for post-processing at a later stage of the system. Practically, segmentation of the region of interest is semi-automatically determined with external peripherals. In the case of ultrasound or tagged MR imaging, tracking of moving signals encoded onto the medical images can be carried out by a motion estimation component to define the wall boundary. The geometrical structure is scaled and displayed via a suitable displaying element. Optionally, other measures such as the velocity of cardiac wall can be computed with a numerical simulation framework using computational fluid dynamics (CFD) and implemented for cardiac flow analysis. Note that the receiver, segmentation, boundary extraction, geometrical surface reconstruction elements forms the main processing system of this framework. The components within this processing system are utilized for post-processing medical images and outputting the geometry of the anatomical structure of interest. Outside of this system are the physical hardware that facilitates the medical scanning, user inputs and display, which we will not explore further. Let us examine each of the components with greater detail in the subsequent sections.

Magnetic resonance imaging is a well-established medical imaging modality, and can be used effectively for imaging the heart and large arteries for cardiovascular study. Diagnostic system for detecting cardiac abnormalities and quantifying the degree of cardiovascular defects has proven to be clinically attractive [25–32]. The information from MRI will have useful future practical benefit for the assessment of cardiac-related failures.

**Fig. 3:**
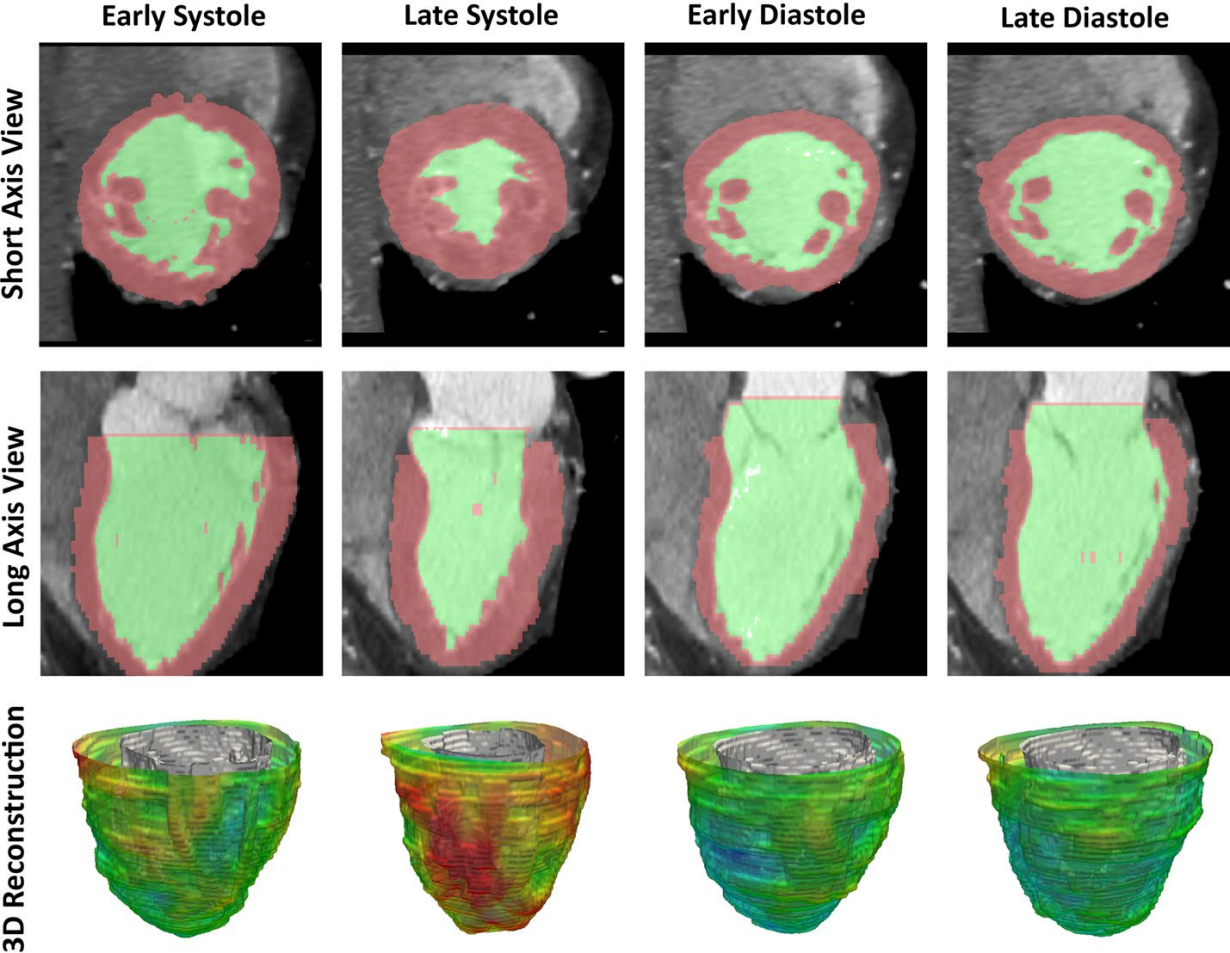
Geometrical reconstruction of left ventricle based on computer tomography. The images depict a short-axis (*top*) and long-axis (*middle*) scanning of the heart. The thickness of left ventricular endocardial and epicardial surfaces are traced with color mapping. Based on the myocardial segmentation, three-dimensional (3-D) reconstructions of the left ventricle (*bottom*) are prepare. The cardiacphases at the early, late diastole and systole are used as the time reference for hemodynamic assessment

Now let us examine a more practical example, in which magnetic resonance imaging (MRI) or computer tomography was performed to study myocardium problems. The images were then used to reconstruct diastolic to systolic LV anatomy (Fig. 3). The parameters of the LV such as regional shape, surface curvedness, wall thickness, and wall stress indices were assessed. In Zhong’s study similar analogy is applied on pre- and post- SVR in the short-axis and long-axis orientations of the heart [14]. Quantification of the infarct, border, and remote zones based on end-diastolic wall thickness was performed. We can then study the blood flow in the heart with this imaging modality. We will elaborate more on this in “Left ventricular computational analysis: to study left ventricular functional performances, and how to maintain its health” section subsequently.

### Reconstructing surface mesh and boundary conditions

After extracting the cardiovascular structure by segmentation, the computational file can be saved as an IGES, STL, or STEP in order to be cross-compatible with a range of 3D modelling and meshing programs that can result in different types of mesh configurations. Typically, the meshing procedure begins by the application of a simple unstructured tetrahedral mesh all over, which produces a single contiguous mesh. However, for easier post-processing of local flow variables, the computational model may be split into smaller sub regions during the CAD surface and volume generation stage, prior to meshing. While the process of sub-dividing the computational model into smaller regions can be performed within some CFD packages, it is not always an easy task, and therefore it is recommended to be performed in CAD packages that have NURBS functionality.

#### Stenosed artery and left ventricle models

The generation of a quality mesh is of extreme importance for obtaining reliable computational solutions. A good quality mesh improves numerical stability, and increases the likelihood of attaining a reliable solution. A mesh can be viewed as a number of smaller mesh or grid cells that overlays an entire domain geometry. In general, the set of fundamental Navier–stokes equations representing the flow physics are applied onto each cell. These equations, which calculate the flow variables in each cell within the domain, are subsequently solved to yield the corresponding *discrete* values of the flow-field variables such as the velocity, pressure, and temperature.

For the meshing of a cardiovascular structure, such as an atherosclerotic artery, surfaces are created and stitched up to create a computational mesh. An initial model with *N* number of cells is created. The original model is refined by cell adaptation techniques that include refining large volume cells, that display high velocity/pressure gradients and near wall refinements. This process is repeated twice, with each repeat producing a model with a higher cell count than the previous model. These models are used in simulation, and outputs such as velocity profiles are compared. Then, the model with the mesh that did not result in a significant difference in flow results presented by the mesh of higher density at its next step is selected for further runs. An example of a rectangular mesh for the left ventricular and corresponding computational geometry is demonstrated by Schenkel et al. [33]. The transformation must be defined, such that there is a one-to-one correspondence between the rectangular mesh in the computational domain and the curvilinear mesh in the physical domain. The algebraic forms of the governing equations for the multiphase problems are carried out in the computational domain which has uniform spacing of $\Delta \xi$ and uniform spacing of Δ*η*. Computed information is then directly fed back to the physical domain via the one-to-one correspondence of grid points. Because of the need to solve the equations in the computational domain, they have to be expressed in terms of curvilinear coordinates rather than Cartesian coordinates, which means that they must be transformed from (*x*, *y*) to $(\xi , \eta )$ as the new independent variables.

#### Aortic dissection and carotid bifurcation models

**Fig. 4:**
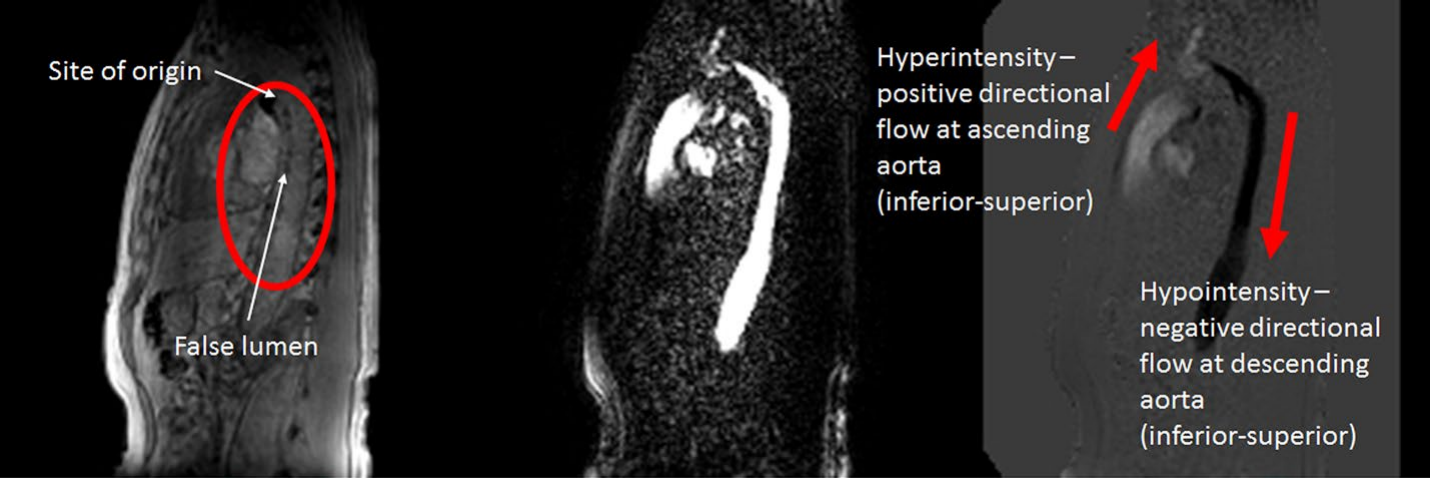
Phase contrast measurement of blood flow in aorta. (*Left*) Structural T1 imaging of a dissected aorta; (*middle*) magnitude image of spin echo signal in phase contrast MRI; (*right*) phase image in phase contrast MRI. The images are captured in sagittal view with inferior-superior direction velocity encoding. In structural image the artery can clearly identified with two separated compartments. The velocity can also encode in anterior-posterior and left–right direction to generate orthogonal velocity vector components. This can be used to demonstrate 3 + 1D flow patterns and indicating the presence of complex flow along the channels of the artery. The reconstructed flow field could also applied as a boundary condition and validation of computerized flow simulations

Scan images based on the velocity-encoded MRI modality, as shown in Fig. 4, can be performed for the cardiovascular diseases such as aortic dissection and carotid artery [34]. VENC MRI enables the direct visualization of flow without the need to carry out high-performance computing of the data for modeling. The intensity of phase image is directly proportional to velocity of the fluid if proper MR pulse sequence is applied. However, it is unable to predict future flow events, such as conditions when the dissected aneurysm in an artery worsens or post-surgical treatment outcomes in the case of surgical reconstruction.

For a realistic simulation of cardiac structures, boundary conditions can be well-established and derived based on measurement of cardiac flow profiles obtained from velocity-encoded imaging modalities by the phase contrast MRI scans. The understanding and derivation of flow properties in such conditions are necessary. The accuracy of the hemodynamics of an isolated patient-specific cardiovascular structure highly depends on the application of the boundary conditions. As such, phase contrast MRI may be used to provide these boundary conditions for the computational model, which also serve the additional purpose of providing a basis for analysis of the flow nature in addition to the computational prediction after that.

**Fig. 5:**
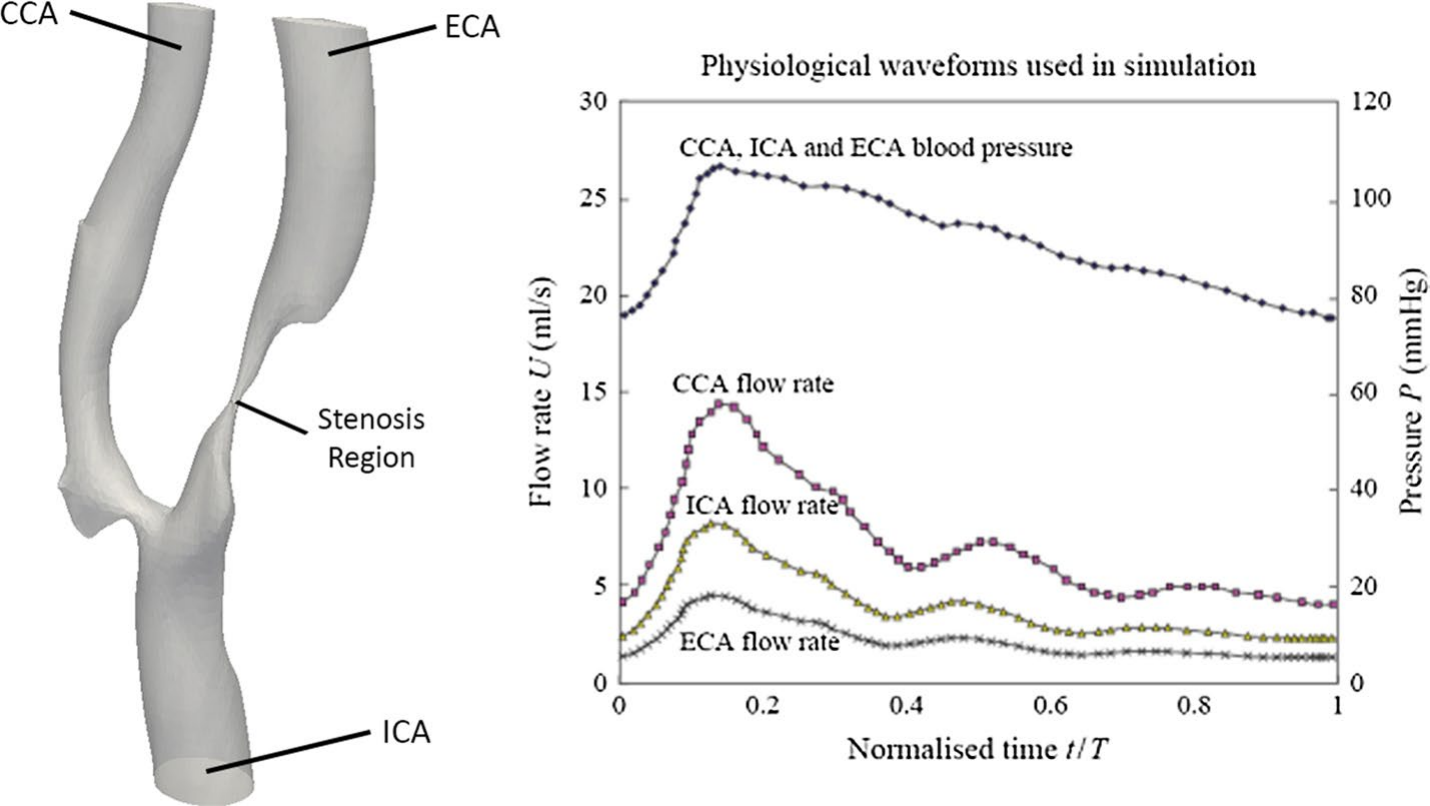
Physiological waveforms used in simulation. This fluid mechanical property, which is based on flow rate, Q (ml/s), serves as the inlet boundary condition for the carotid artery used in CFD simulation. For the cardiac cycle based on duration of *T*, the systolic phase (at *t* = 0.1*T*), peak phase (at *t* = 0.2*T*) and diastolic phase (at *t* = 0.5*T*) are characteristic of the waveform

Specifically, let us examine the group mean blood flow waveform as shown by Fig. 5, which is based on flow rate, *Q* (ml/s), for the ICA, ECA and CCA [35], which was introduced as the flow boundary condition for the carotid artery object of interest. Then, experimentally derived volumetric flow rates can be set at one inlet and two outlets.

#### Intra-ventricular flow model

**Fig. 6:**
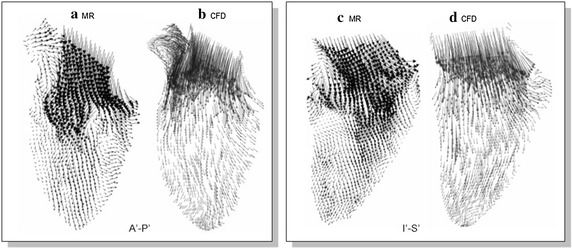
Flow fields of MR velocity imaging and CFD simulation. A 2D section of the velocity fields by the MRI modality and CFD simulation are displayed to characterize the flow within the left ventricle. The influxes of blood into the heart chamber as displayed by the two techniques generally possess the same kind of swirl nature. (Images from [36])

In the context of intraventricular flow, the cardiac flow fields of a subject may also be fully measured and compared with the computational model as shown in Fig. 6. It may be worthwhile noting that the measured flow patterns can be used as a gauge for checking if the simulation settings are valid. As such, in terms of validating the CFD results, imaging modalities such as MRI can be effectively used. Pairs of flow fields generated by CFD simulation and MRI experimentation can be compared quantitatively for the purpose of establishing the computational platform properly prior to further analysis. The two sets of results constitute an overall topological structure. The flow rates of the hemodynamic structure can be determined and used as boundary conditions for the simulation. It is worthwhile noting that the inflow boundary conditions play an important role in developing reproducible simulations, because the computational model is extremely sensitive to these imposed conditions [33, 36].

### Hemodynamics health indicators of flow through a carotid bifurcation model

Velocity vector plot and axial profiles arising from computational hemodynamics can be used to assess influence of arterial stenosis on the flow through a carotid bifurcation model. The flow patterns within the carotid artery due to the geometry of the bifurcation can be assessed by vector associated streamlines as well as flow profile plots [9].

**Fig. 7:**
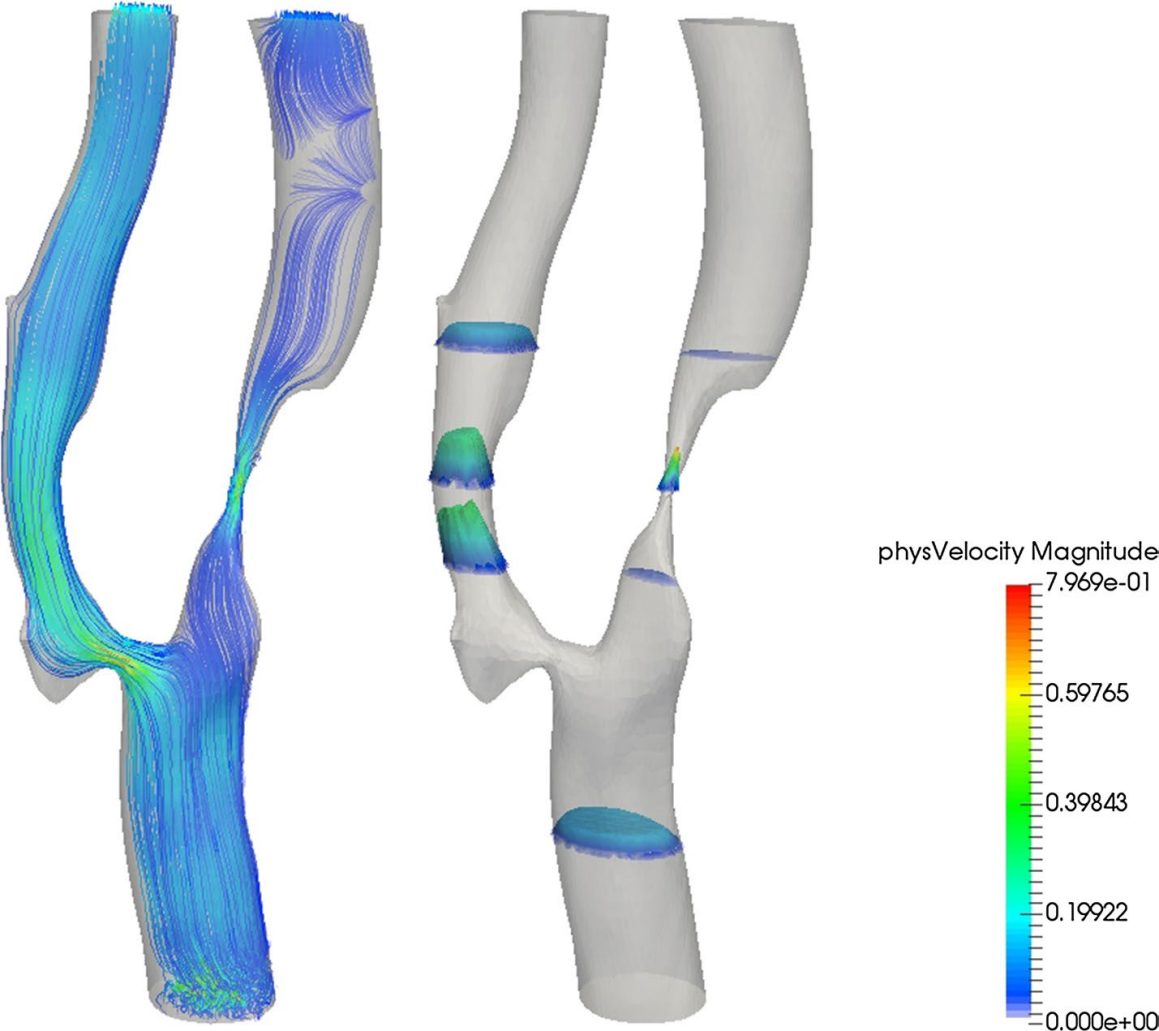
Velocity streamline plots and axial velocity profile of flow in atherosclerotic artery. *Left* velocity streamline plots of carotid bifurcation in the branching plane at t/T = 0.6 (systolic acceleration phase); *right* the axial velocity profile of flow through the four sections of the carotid bifurcation. These results were prepared by CFD simulation using high-performance computing. Magnitude of the relevant velocity is rendered by *color* coding scheme

As seen in Fig. 7, the axial velocity profiles in the bifurcation plane have a high degree of skewness near the bifurcation region. Downstream of the bifurcation or flow divider and along the ECA, the axial flow accelerates due to the reduction in cross-sectional flow area as a result of the stenosis. On the other hand, note the lower axial velocity at the sinus bulb of the carotid artery. It is worthwhile mentioning that such consistently slow moving flow at this region may give rise to a higher susceptibility of deposits [37]. The stenosis in the artery branch affects the flow field more in the ICA than in the ECA. At the ECA, the flow velocity is more consistent in terms of velocity profile upstream of the artery branch. The presence of the stenosis in the ICA does not affect the flow field in the ICA significantly due to its different branching.

From the velocity vector plots and axial velocity profiles, we are able to examine regions of flow separations, accelerating and decelerating flows, as well as their transient magnitudes. From the simulation, we are only able to detect the flow and how it affects the vessel wall, specifically luminal diameter or stenosis, plaque volume and wall thickness. However, we are unable to assess the health conditions of atherosclerosis from the flow velocity information. As such, a more precise indicator should be deployed in order to assess the disease more appropriately. Together with medical imaging, we can then see a whole greater picture. By seeing all these, the doctor can identify the high risk plaques that are vulnerable to rupture and thrombosis better.

For this purpose, let us discuss some parameters commonly used to assess blood flow. Certain hemodynamics parameters require a clear definition prior to performing simulation of the blood in the circulatory system comprising the arteries and heart.

Resistance of blood to deformation under shear stress causes viscosity. Practically, we can describe its internal resistance to flow as a form of fluid ‘friction’. Binding of the molecules pertaining to the fluid is responsible for this viscosity. Mathematically, viscosity is defined as the ratio of the shear stress to the velocity gradient, which can be represented as the shear rate.

Most fluids approximate Newtonian fluids, resulting in a constant viscosity. Nevertheless, blood, which consists of plasma, blood cells and other material carried throughout the bloodstream, tends to cause blood to become non-Newtonian due to the quantity of particles within the plasma. In fact, the blood viscosity changes with the shear rate of the flow. When the shear rate is sufficiently high, blood flow exhibits Newtonian flow behaviour. Realistically, under normal conditions it is not viable to ignore the non-Newtonian behaviour of the fluid.

In fluid flow that is incompressible and under a steady flow field, the shear rate of strain of a material fluid element is defined as the rate of decrease of the angle formed by two mutually perpendicular lines on the element. As such, the shear strain rate $\dot{\gamma }$ is proportional to the rate of decrease of axial velocity *v*_*z*_ along the arterial radius:

1 $$\dot{\gamma } = - \frac{{dv_{z} }}{dr}$$

The rate of change in velocity along the radial section from the wall to the centre of the vessel, which was previously defined as the shear strain rate, is proportional to the wall shear stress. The mechanical characteristics of the flow can be described by the Poiseuille’s law model, which defines a linear relationship between the shear stress *τ* and strain components with the viscosity *μ* as its gradient. Therefore, the equation for wall shear stress is given by:

2 $$\tau = \mu \dot{\gamma }$$

Blood flows along curved arteries with complex flow dynamics, such as variable axial velocities along the radial section [38]. In the case of carotid arteries, the flow velocity varies in magnitude at regions close to the other wall and near the bifurcation [39]. This complex phenomenon is responsible for the time-dependent oscillatory wall shear stress distribution throughout the blood vessel.

Three commonly adopted flow indicators to evaluate the total shear stress exerted on the wall throughout a cardiac cycle are the time-averaged wall shear stress (TAWSS), the oscillatory shear index (OSI), and the relative residence time (RRT), which are presented in the following equations:

3 $${\text{TAWSS}} = \frac{1}{T}\int_{0}^{T} {\left| {\tau_{w} } \right|} dt,$$

4 $${\text{OSI}} = \frac{1}{2}\left[ {1 - \frac{{\left| {\frac{1}{T}\int_{0}^{T} {\tau_{w} } dt} \right|}}{{\frac{1}{T}\int_{0}^{T} {\left| {\tau_{w} } \right|} dt}}} \right],$$

5 $${\text{RRT}} = \frac{1}{{\left( {1 - 2 \times {\text{OSI}}} \right) \times {\text{TAWSS}}}},$$

where *T* is a cardiac cycle period and *τ*_*w*_ is the instantaneous wall shear stress. The atheroprotective effect of the endothelial cells is influenced by these hemodynamic indicators. Low TAWSS values (lower than 0.4 Pa) [40], high OSI (higher than 0.5) [41–43] and high RRT (higher than 10 m^2^/N) [13, 44] are known to promote an atherogenic endothelial phenotype, while abnormally high TAWSS (higher than 40 Pa) values can cause direct endothelial injury and increase the risk of thrombosis [40, 45].

According to the thresholds of the local flow indicators, the outer-wall of the ICA at the bifurcation territory of the healthy carotid case study is where the low TAWSS, high oscillatory shear and long relative resident time exist. Therefore, it is a vulnerable site for atherosclerosis in the long term. The apex of the divider-wall of the bifurcation experiences the maximum TAWSS, as compared to the remaining portion of the healthy carotid bifurcation. Because this peak TAWSS value is much lower than the threshold, this site is still risk-free of getting direct endothelial injury from the blood flow.

By referring to the values of TAWSS and OSI, the prediction of the degree of atheroprotectiveness or atherosusceptibility can be made. The fraction of luminal surface that is non-atheroprotective is more prone to atherogenesis. The studied diseased carotid bifurcation experiences a high TAWSS at the throat of the ICA stenosis, which gives a warning of stroke, and the blood transportation is also disturbed as the flow division value deviates from normal situation constantly. In such a case, a vascular intervention is required.

From the physiological perspective, the carotid bulb is a common atheroprotective location for both the healthy case study and the diseased case study, because of the commonly formed disturbed flow at this region. Based on the arterial hemodynamics indicators, a strategy for cardiac management of the atherosclerotic patient can be devised based on the case study report of the diseased artery. Diagnosis via cardiovascular indicators is transferred electronically or in hard copy format to medical professionals for advice [46, 47].

### Left ventricular computational analysis: to study left ventricular functional performances, and how to maintain its health

Computational medical imaging and fluid dynamics analysis can be applied in the context of evaluating the performance of heart pumping, such as its efficiency in contraction and dilation throughout the cardiac cycle. Let us study what happens to a cardiomyopathy left ventricle, which has reduced contractile capacity for pumping out adequate cardiac output (CO). A manifestation of cardiomyopathy and its decreased contractile capacity is the inability of the LV to retain its compact systolic curved shape. In other words, a cardiomyopathic left ventricle undergoes remodeling, its curvedness index decreases, and it becomes more spherically shaped—which further reduces its contractility index (as given by ${\text{d}\upsigma^{*}/\text{dt}}_{{\text{max}}} \text{ = 1}{.5 \times {\text{dv}}/{\text{dt}}}_{{\text{max}}} \text{/V}_{\text{m}}$). As LV function deteriorates, the symptoms of heart failure (HF) become evident.

In these cardiomyopathy left ventricles, computational fluid dynamics can provide quantitative analysis of intra-LV blood flow outcomes of remodeled LVs, and even enable computational simulation of surgical ventricular restoration (SVR) of cardiomyopathy hearts. In other words, prior to carrying out SVR, we can simulate different measures of surgical truncation of the LV, determine the values of the truncated LV’s contractility index and analyse their intra-LV blood flow distributions. We can then arrive at what SVR measure provides the optimum value of the contractility index and optimum intra-LV blood flow for minimal truncation. This can facilitate preoperative modeling for patients to achieve optimized post-SVR flow performance [15]. By knowledge of these remodeled LV’s curvedness and contractility parameters, we can then predict and assess restoration of the heart pumping efficiency based on the information provided by CFD [48, 49].

The nature of intra-LV fluid flow can be characterized by means of the fluid dynamics parameters of the Womersley number, *Wo*, and the Reynolds Number, *Re*, as well as the exchange transfusion that is denoted by *M*, which represents the remaining fractional blood volume in the ventricle after an arbitrary number of cardiac cycles, *n* [15].

6 $$M = \left( {\text{1} - EF} \right)^{n}$$

As can be observed from the computed data, the exchange transfusion, *M*, shows consistently higher value for a patient as compared to a healthy normal subject, which indicates that more of the blood volume remained in the left ventricle at the end of every cycle. The intraventricular condition of a patient is such that it has a much less efficient wash-out due to the greater residual volume in the left ventricle at the end of ejection. Furthermore, the *Wo* is demonstrated to be moderately lower for such a patient as compared to the healthy subject, and *Re* is in the order of one magnitude lower than in a healthy subject [15].

It may be worthwhile mentioning here that traditional diagnosis of left ventricular (LV) dysfunction is based on cineangiograms, ultrasound monitoring or MRI scans of LV wall motion. Observation of the improvement in wall motion leading to a higher ejection-fraction after administration of myocardial vasodilators can determine if coronary bypass surgery can be beneficial. In this regard, flow related quantification parameters can be determined: (1) end-diastolic volume (EDV) and pressure (EDP), (2) stroke volume (SV) and stroke work (SW), (3) LV resistance-to-filling and contractility, (4) ejection fraction (EF = SW/EDV), (5) maximal rate of change of pressure-normalized stress, and (6) regional function in terms of change of the endocardial surface area (S) such that ΔS = (S_ED_ S_ES_)/S_ED_ × 100% for end diastole (ED) to end systole (ES) [50].

Furthermore, in the LVs, there is also reduced contractility; so we also need to provide an index for cardiac contractility, in terms of maximal rate-of-change of normalized systolic wall stress, *dσ* ^*^/*dt*_*max*_, and its decrease in an infarcted LV progressing to heart failure [51]. This contractility index is based on the evidence that in systole it is the generation of LV wall stress that gives rise to increase of LV pressure. Hence it is rational to base the contractility index on the rate of increase of LV wall stress normalized with respect to the LV pressure—which makes the contractility index to be non-invasively determined.

These above mentioned parameters can assess the heart performance [14, 52]. For example, we can determine (1) how decreasing resistance-to-filling during diastole and a higher intra-ventricular pressure gradient during systole, and the ratio of stroke work to end-diastolic volume will improve ventricular pumping efficiency, (2) LV systolic performance in terms of stroke work ($SW = SV \times \bar{P}_{a}$), whereby $\bar{P}_{a}$ denotes the mean arterial pressure, and LV systolic function in terms of the EF, and (3) the contractile capacity of the LV in terms of the maximal rate of change of pressure-normalized stress (dσ*/dt_max_ = 1.5 × dV/dt_max_/V_m_, given that dV/dt is the first derivative of the volume and V_m_ is the myocardium volume at end diastole [50], (4) the stroke work index, defined as (SW = SW/EDV) as a measure of LV systolic function. Another important parameter to look at is the global shape of the LV, which is characterized by a sphericity index defined as the ratio of the short axis to the long axis [53, 54]. As such, these performance parameters, in addition to the fluid mechanical parameters determined by the computer simulation, can be used hand-in-hand to provide the health measure of the cardiac chamber.

Let us elaborate on the use of some of these indices by providing the below (i) Table 1 to demonstrate how surgical ventricular restoration improves LV sphericity index and contractility.

**Table 1:** Patients’ data pre- and post-SVR, showing improved contractility after SVR

Variables	Pre SVR (n = 40)	Post SVR (n = 40)
Cardiac index (L/min/m^2^)	2.84 ± 0.74	2.59 ± 0.74
Mean arterial pressure (mmHg)	85 ± 14	84 ± 8
Systolic blood pressure (mmHg)	115 ± 20	113 ± 10
Diastolic blood pressure (mmHg)	71 ± 12	70 ± 8
End diastolic volume index (ml/m^2^)	156 ± 39	110 ± 33*
End systolic volume index (ml/m^2^)	117 ± 39	77 ± 31*
Stroke volume index (ml/m^2^)	39 ± 9	33 ± 8*
Left ventricular ejection fraction (%)	26 ± 7	31 ± 10*
LV mass index (g/m^2^)	112 ± 25	101 ± 23*
End-diastolic long axis, BA_ed _(cm)	10.89 ± 1.16	8.31 ± 1.00*
End-diastolic short axis, AP_ed_ (cm)	7.00 ± 0.80	6.64 ± 0.78*
End-systolic long axis, BA_es_ (cm)	10.37 ± 1.20	7.87 ± 1.05*
End-systolic short axis, AP_es_ (cm)	5.86 ± 0.98	5.23 ± 1.06*
End-diastolic sphericity Index, SI_ed_	0.65 ± 0.087	0.81 ± 0.11*
End-systolic sphericity index, SI_es_	0.57 ± 0.094	0.67 ± 0.13*
Difference between end-diastolic and end-systolic sphericity index, SI_ed_ − SI_es_	0.077 ± 0.043	0.14 ± 0.059*
Long axis shortening (%)	4.8 ± 3.6	5.4 ± 4.4
Short axis shortening (%)	16.4 ± 6.8	22 ± 9.7*
*dV/dt*_*max*_ (ml/s)	364 ± 83	401 ± 81*
Pressure normalized wall stress	4.30 ± 0.95	3.31 ± 0.75*
Stroke work (mmHg l)	6.61 ± 1.96	5.46 ± 1.64*
dσ*/dt_*max*_ (s^−1^)	2.69 ± 0.74	3.23 ± 0.73*

Values are mean ± SD

* p < 0.05

## Combined contribution of medical imaging and computational hemodynamics to cardiovascular diagnostics

In general, cardiac medical imaging has been widely utilised for imaging patients with various cardiac conditions, such as diseases of the aorta [25, 26, 55], aneurysm [27, 28], and human hearts with atrial and ventricular septal aneurysm or defect [29–32]. However, traditionally, only anatomical information of the cardiovascular compartments or structures has been used for diagnosis, until the development of phase contrast MRI that is even able to provide cardiac flow quantification. On the numerical modeling front, CFD is able to provide simulation of various scenarios or conditions, such as pre- and post- surgical treatment, whereby the actual surgical procedure is benefitted by the pre-surgical simulation.

We can summarize the clinical applications of medical imaging in the cardiovascular system for which the following medical conditions are assessed [56]: (1) pericardial disease; (2) congenital heart disease; (3) aortic arch heart disease; (4) acquired heart disease; (5) cardiac transplantation; (6) atrial and ventricular septal defects; (7) valve regurgitation; (8) aneurysms; and (9) coarctation of the aorta. The quantification of flow characteristics within the heart and arteries provides vital information to cardiologists, who are interested in a range of problems from blood flow hemodynamics to myocardial biological processes in the heart. Intra-LV flow inefficiencies such as whirlpools or swirling structures, as well as turbulence can be examined, and their existence can ultimately be linked to the operation of a number of cardiac structures (such as defective heart valves) influencing flow in the heart. This has potential applications for identifying risks for heart failures, stroke and plaque vulnerability, and may match the current state of art technologies in terms of cardiac analysis.

### Computational intra-LV blood flow patterns in normal subjects and heart failure patients

**Fig. 8:**
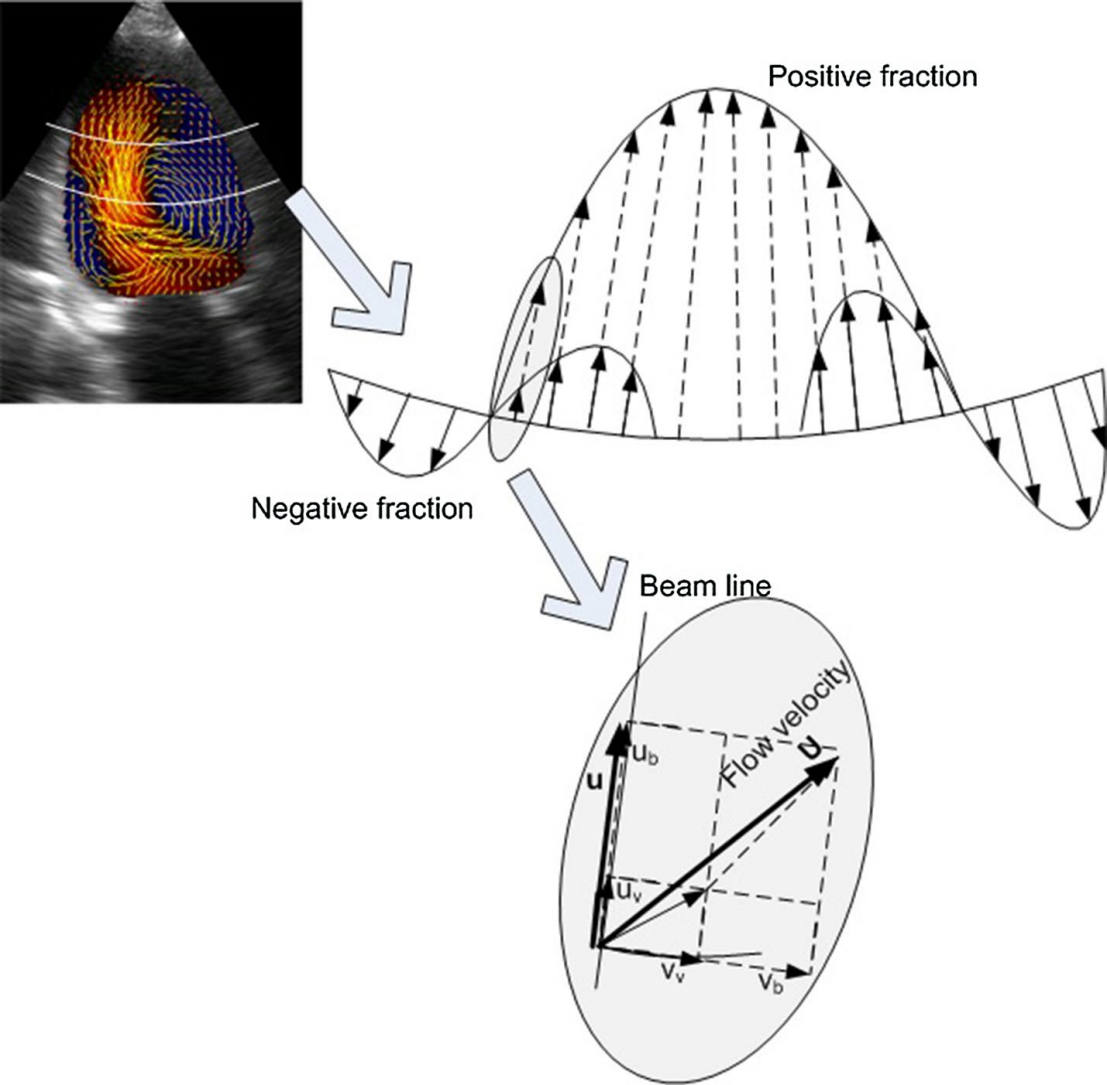
Velocity generated by VFM along an arc at each echo depth with a combination of single laminar flow and vortex flows. Colour Doppler flow data are separated into basic and vortex flow components so that vortex flow component is bilaterally symmetrical on each arc. At a given pixel, colour Doppler velocity u along the beam line is a sum of its vortex flow component $u_{v}$ and basic flow component $u_{b}$. The vortex flow component consists of colour Doppler velocity $u_{v}$ and radial velocity $v_{v}$. Likewise, the basic flow component consists of colour Doppler velocity $u_{b}$ and radial velocity $v_{b}$. Flow vector is the sum of flow vectors of basic and vortex flow components

The importance of intra-LV blood flow patterns is that they can be looked upon as functional outcomes of the heart, and hence provide us quantifiable basis of assessment of heart failures with normal and reduced ejection fractions. We now present the determination of intra-LV blood flow patterns, by use of the ventricular flow mapping (VFM) analysis package (DAS-RS1) [57]. In this technique, colour Doppler velocity (axial velocity, **u**) profile is analysed across an arc at each depth, as shown in Fig. 8. The Doppler velocity** u** is composed of basic non-vortical laminar flow (**u**_**b**_) and vortex flow (**u**_**v**_) components. If the Doppler velocity profile on the arc has both negative and positive fractions, it is considered to be a combination of non-vortical and vortical laminar flows. The vortex feature is assumed to be bilaterally symmetric so that the negative and positive components of** u**_**v**_ perpendicular to the arc negate each other (Fig. 7). As illustrated in Fig. 8, the flow velocity components** u**_**b**_ and ${\textbf{u}}_{\textbf{v}}$ are in the Doppler beam direction (axial), while ${\textbf{v}}_{\textbf{b}}$ and ${\textbf{v}}_{\textbf{v}}$ are in the direction perpendicular to the Doppler beam (radial).

### Importance of integrated platform combining computational imaging and hemodynamics

It is important to note that vessel or heart chamber imaging and visualization is not sufficient as a stand-alone information provider of the cardiac health assessment. The important hemodynamic parameters or indicators affecting cardiovascular health performance discussed in this paper can be coupled along with the information based on heart imaging to serve as a more potentially reliable diagnostic system for the assessment of heart disease and as a practical tool for physiological analysis. For example, in addition to the evaluation of atherosclerosis and heart pumping performance as discussed in this paper, hemodynamics performance indicators also have the potential applications for identifying risks after heart valve implant as well as determining the degree of atrial or ventricular septal defects. The simulation may also be used to examine the growth effect of cardiac tissue into the mesh of surgically implanted device from the fluid mechanical perspective, and hemodynamics indicators such as vorticity can be designed to assess its health performance [46].

Quantification of cardiac health that uses an integrated image processing and high-performance computing approach can be used on a patient-specific basis without the need for additional diagnostics or in vivo procedures, and thereby making it attractive for future clinical practice. The healthcare industry comprising of hospitals, medical institutes and universities will benefit by using the simulated hemodynamics performance indicators for evaluation of cardiac health, and using the hemodynamic flow fields as an assessment for analysis of flow phenomena to assess the impact of cardiac pathology. Manufacturers of medical imaging machines can also beneficially incorporate such computer visualization techniques into their imaging systems. The imaging data can be transferred to dedicated computer laboratories with high-performance computing facilities, to generate the appropriate hemodynamics health indicators. This presents a viable integrated platform for the purpose of flow analysis and virtual intervention outcome prediction for vascular diseases. Medical organizations (from the cardiac discipline) can employ this platform to assess the health of human heart and arteries so that appropriate medical action can be taken.

## Summary of review

Being at the cutting edge of medical science, the recent developments in the fields of medical imaging modalities have given new dimensions to our understanding of the human cardiovascular system. However, the potential of using functional health indicators in terms of the hemodynamics within the heart still has some unexplored opportunities for cardiac health diagnostics. In this paper, we have shown that this concept can be integrated into the present medical image diagnostics by well-established computational modeling for the determination of appropriate hemodynamics indicators. Cardiac flow analysis contributes to the development of the framework behind the operation of future flow simulation or mapping systems using high-performance computing. This type of system will lead to a new perspective on cardiovascular performance, risk and health, which can be obtained by using insights from the combined field of medical imaging and computational hemodynamic modelling. Further patient-specific analyses involving the combined field of medical imaging and visualization and high-performance computing will significantly contribute towards providing more reliable and precise cardiovascular health performance indicators and associated benefits.

Medical imaging-based diagnostics integrated with high-performance computing will ultimately have a big impact on more precise medical diagnostics. The computational flow indicators and visualization to couple with anatomical details will constitute a significant forefront in technological development of the next era. The introduction of new diagnostic procedures for the evaluation of heart defects, as well as the capability of the research to identify and quantify flow phenomena through it will potentially result in useful clinical information to provide cardiologists a leading edge in saving patients.

## Appendix

See Table 2.

**Table 2:** Details of procedures used in medical imaging for hemodynamic analysis and diagnostics

Geometrical reconstruction		
Medical imaging	Voxel reconstruction	3D contour
Ultrasound (medical imaging using vibration/sound of ultrasonic frequency)	Draw boundary curves in the voxel space	Region-based segmentation methods use an algorithm that searches for similar feature e.g. texture pattern and brightness
MRI (uses magnetic field and radio waves)	Connect these points with spline curves and digitize	Divide image into region and check similarity among pixels. Continue dividing for similarity below threshold
PET [by injecting, swallowing or inhaling radioactive drug (tracer)]	Fill the inner part of the boundary	Merge regions with similar features
CT (combined X-ray images from different angles)	Execute the same procedure to X and Y planes	Repeat these two steps until no more merging or splitting is possible
X-ray (high frequency and energy electromagnetic radiation)	Smooth, sum up 3D spaces and cut by threshold values	Active contour combines global and local image segmentation methods
ECG (measuring electrical activity of the hearts using electrodes)		
fMRI (using MRI and associated changes with blood flow)		

3D modelling		
Volumetric reconstruction	Volume rendering	3D image modelling
Define voxel space	Display a 2D projection of a 3D discretely sampled data set, typically a 3D scalar field	Mathematical representation development of an object’s 3D via specialized software that results in a 3D model
Reconstruct to produce transparent or opaque voxel		
Curved inconsistent voxel		

3D modelling		
Contour based reconstruction	Surface rendering	3D surface shading
Binary image of closed curves represents each contour and produces a smooth 3D mesh output	The process of generating an image from a 2D or 3D model (or a scene file collectively) using a specialised computer program	Altering the color of an object/surface/polygon in the 3D scene, based on:
The individual slices of images are stacked to produce a 3D dataset		Its angle to lights
Slice number and contour pixels’ indices convert the contour data into a set of points for reconstruction process		Its distance from lights
		Shading is performed during rendering

Simulation		
Import of model and mesh definition	Setting of boundary conditions	Definition of relevant models using computational fluid dynamics
Import boundary mesh	Create finite element function	Achieve multi-scale modelling by coupling (1D-3D NS and IFS)
Quality check of boundary mesh	Define trial and test function (function space)	Branch of fluid mechanics that uses numerical analysis and algorithms to solve and analyze fluid flows problem
Volume mesh generation	Specify boundary condition and subdomain indicator (information stored in mesh)	Computers perform the calculations to simulate liquids and gases interaction with surfaces defined by boundary conditions
Mesh refinement performance	Constitutive laws of materials (blood and walls). Involved flow governing parameters and blood flow pattern	High-performance computing to deal with the high density grid resolution and deal with large file sizes
Volume mesh quality inspection		
Volume mesh save		
Align the grid lines of mesh with the flow		
Assign high mesh density to capture all relevant flow features and high resolution mesh adjacent to the wall to resolve boundary layer flow		

Medical application	
Model processing and analysis	Surgical assisted application
Breakdown of the phases of a process to convey the inputs, outputs, and operations of each phase	Set of methods and surgical concept that use computer technology for planning, guiding or performing surgical interventions

## Abbreviations

LV: left ventricle
WSS: wall shear stress
TAWSS: time-averaged wall shear stress
US: ultrasound
MRI: magnetic resonance imaging
CT: computed tomographic
OSI: oscillatory shear index
SVR: surgical ventricular restoration
VENC: velocity-encoded
PC-MRI: phase contrast magnetic resonance imaging
CFD: computational fluid dynamics
UTS: ultrasound
CO: cardiac output
HF: heart failure
EDV: end-diastolic volume
EDP: end-diastolic pressure
SV: stroke volume
SW: stroke work
EF: ejection fraction
ES: end systole
VFM: ventricular flow mapping
